# Maxillary Osteonecrosis Related with Herpes Zoster: A Case Report and Review of the Literature

**DOI:** 10.3390/medicina60060883

**Published:** 2024-05-28

**Authors:** Kwan-Soo Park

**Affiliations:** Department of Oral and Maxillofacial Surgery, Inje University Sanggye-Paik Hospital, Seoul 01757, Republic of Korea; OMS_kspark@paik.ac.kr

**Keywords:** herpes zoster, varicella-zoster virus, osteonecrosis, tooth exfoliation, trigeminal nerve

## Abstract

Osteonecrosis of the jaw (ONJ) can occur through various mechanisms including radiation, medication, and viral infections such as herpes zoster. Although herpes zoster is a varicella-zoster virus infection that can affect the trigeminal nerve, it rarely causes oral complications. The author reports a rare case of herpes zoster-related ONJ, followed by a review of the relevant literature pertaining to herpes zoster-related oral complications, including ONJ. A 73-year-old woman presented with a scarred skin lesion on her left midface with an exposed alveolar bone of the left maxilla. Based on her medical records, she received a diagnosis and treatment for herpes zoster six months prior and experienced a few teeth loss in the left maxilla following a fall preceding the onset of herpes zoster. Sequestrectomy of the left maxilla was performed and ONJ was diagnosed. The operative site recovered favorably. Although unusual, several cases of localized extensive ONJ in herpes zoster-infected patients have been reported. This case illustrates the possibility of a rare occurrence of unilateral widespread osteonecrosis of the jaw (ONJ) even in the maxilla associated with herpes zoster. The exact mechanism has not been elucidated; nevertheless, surgeons should consider the possibility of oral and dental complications, including ONJ, related to a history of herpes zoster.

## 1. Introduction

Herpes zoster is caused by the varicella-zoster virus (VZV), also known as human herpes virus 3 [1]. Primary infection causes varicella (chickenpox), after which the virus becomes latent in ganglionic neurons along the entire neuraxis. With advancing age or immunosuppression, cell-mediated immunity to VZV declines and the virus reactivates to cause zoster (shingles), which can occur anywhere on the body [2]. Although relatively infrequent, oral and/or dental complications, including osteonecrosis of the jaw (ONJ), can cause severe problems.

Recently, the focus on ONJ has been intensively centered on “medication-related ONJ” (MRONJ), and the term is sometimes used synonymously [3]. It has been established that some drugs, including bisphosphonates, denosumab, and antiangiogenic medications, induce MRONJ. However, necrosis of the jawbones is also associated with various other factors [3]. Osteonecrosis is mostly caused by the above-mentioned medications and radiotherapy, but also occurs in patients who acquire infections, or experience trauma, bone disease, or vascular or circulatory dysfunction. Insufficient blood supply to bony tissues caused by systemic diseases, such as severe malnutrition, diabetes, leukemia, alcohol abuse, AIDS, agranulocytosis, syphilis, or Paget’s disease, leads to significantly higher risks for osteonecrosis. The first case of bone changes associated with herpes zoster was reported by Rose in 1908 [4]. Since then, although rare, there have been several reports in the literature describing herpes zoster-related osteonecrosis. The exact mechanism of herpes zoster-related ONJ remains unclear; similarly, the pathophysiology of MRONJ is not completely understood [5,6].

The present report describes a case of maxillary osteonecrosis related to herpes zoster infection in the absence of systemic disease. While various dental complications associated with herpes zoster, such as tooth exfoliation, periodontitis, devitalized pulps, periapical lesions, and root resorption, have been documented, one of the most serious complications is osteonecrosis of the jaw (ONJ) [7,8]. The ensuing discussion briefly reviews the relevant literature pertaining to herpes zoster-related dental complications, including ONJ.

## 2. Case Report

**Figure 1:**
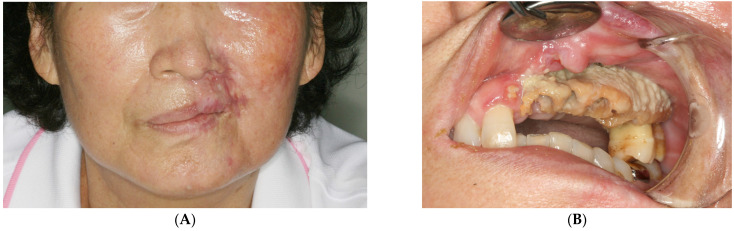
Initial clinical examination. (**A**) A scarred skin lesion on the left infraorbital area is seen. (**B**) Exposed alveolar bone is observed at the left maxilla.

A 73-year-old woman fell and lost a few of her left maxillary teeth. A month or two later, she acquired herpes zoster (shingles) with severe pain on the left side of her face and mild limitations in mouth opening, and was admitted to the hospital for a few days to relieve symptoms. This medical history was obtained from the medical records of another hospital where she had been treated for herpes zoster. Six months later, she was referred to the author’s department with the chief complaint of missing teeth and left gingival pain. Her account indicated that she lost a few teeth without facial bone fracture as a consequence of a fall and subsequently lost more teeth following the treatment of herpes zoster. She had a scarred skin lesion on the left infraorbital area (Figure 1A), with exposed alveolar bone observed at the left maxilla (Figure 1B). Teeth #21, 22, 23, 24, 25, and 28 were exfoliated, and the exposed bone was necrotized with remaining teeth #26 and 27. She had no other systemic disease, except for mild hypertension without symptoms. No history of medication or radiotherapy known to cause osteonecrosis of the jaw was found.

**Figure 2:**
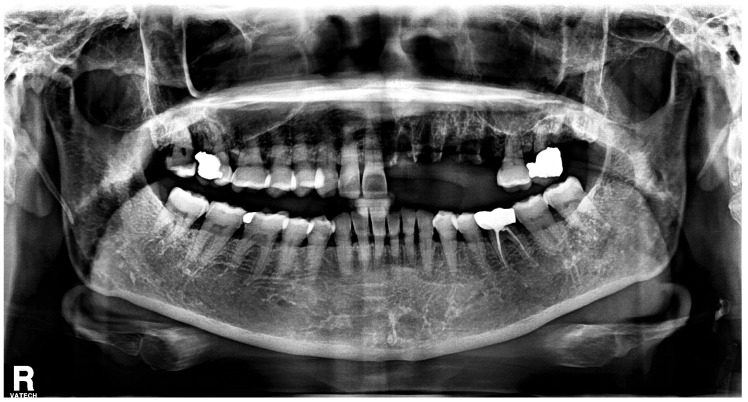
Preoperative panoramic radiograph shows multiple teeth loss and changed trabecular pattern on the left side of maxilla.

**Figure 3:**
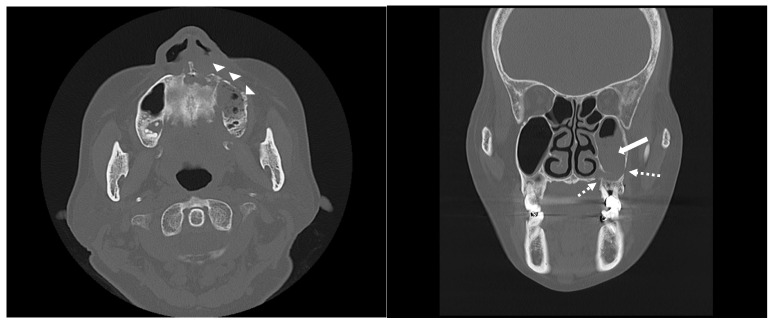
Axial (**left**) and coronal (**right**) CT images reveal pathological fracture (dotted arrows) and ill-defined osteolytic lesion (arrow heads) with maxillary sinusitis (arrow) limited to the left side of maxilla.

**Figure 4:**
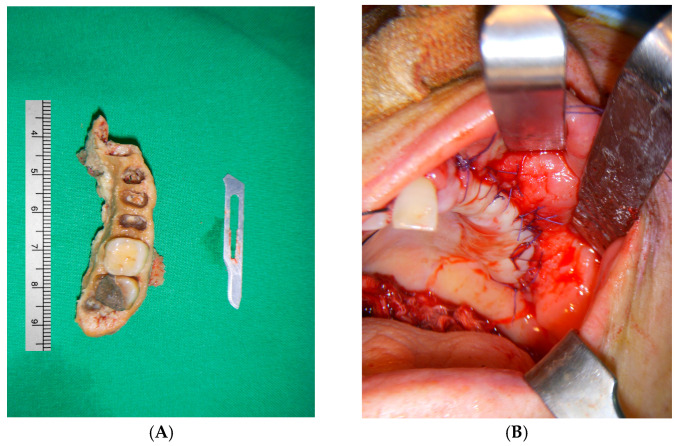
Intraoperative clinical photos. (**A**) Resected necrotic bone. (**B**) Primary closure of operation site was achieved.

**Figure 5:**
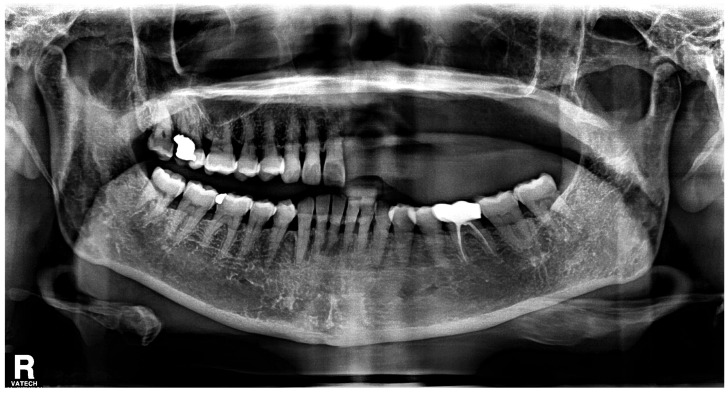
3 months post-operative panoramic radiograph shows no definitive abnormal finding.

With the exception of mild elevation of leukocyte and C-reactive protein levels, all laboratory investigations were within normal limits. An ill-defined osteolytic lesion and abnormally changed trabecular pattern were limited to the left maxilla, and multiple tooth loss with left maxillary sinusitis and pathologic fracture of the left maxilla were observed on panoramic view and computed tomography (CT) imaging (Figure 2 and Figure 3). Based on these clinical and radiological findings, a tentative preoperative diagnosis of herpes zoster-related osteonecrosis of the upper jaw was made. Sequestrectomy of the left maxilla was performed under general anesthesia. Peripheral bone was sufficiently ground using a bur until the sound bone was exposed, with primary closure achieved by gingival coverage (Figure 4). Biopsy revealed acute and chronic inflammatory tissues with bacterial colonies detected on the necrotized bone. Preoperative intravenous antibiotics were given 1 h before surgery. Postoperatively, intravenous antibiotics were given for three days. At the first follow-up 2 weeks after surgery, only mild dehiscence less than 5 mm in length was observed at the suture site without any symptoms, which recovered well with conservative treatment including antibiotics and 0.12% chlorhexidine oral rinse for 2 weeks without additional surgery. 3 months post-operative panoramic radiograph showed no definitive abnormal finding (Figure 5). The resected area was recommended to be restored with a partial denture and she was transferred to a private dental clinic.

## 3. Discussion

Herpes zoster (shingles) is caused by the reactivation of the dormant VZV in the sensory ganglia after an initial varicella (chickenpox) outbreak [9]. VZV establishes latency in ganglionic neurons, and reactivation of viral replication and spread of the virus to the skin innervated by these neurons causes zoster [10]. The thoracic region is most commonly affected (>50%), followed by the face and cervical and lumbosacral regions [2]. Among the cranial nerves affected by herpes zoster infection, the trigeminal nerve is the most commonly affected (18.5 to 22% of cases), followed by the glossopharyngeal and hypoglossal nerves [11]. Among the trigeminal nerves, the ophthalmic nerve is most commonly affected [12]. To the author’s knowledge, there are no published studies reporting differences in infection rates between the maxillary and mandibular branches. Commonly known complications of VZV include vasculopathy, meningoencephalitis, postherpetic neuralgia, myelopathy, and ocular disease [1,2]. There are various risk factors for complications of VZV infections [10]. Risk factors for developing herpes zoster include immunosuppression, older age, malignancy, chronic kidney or lung disease, disorders of cell-mediated immunity such as HIV infection, and a family history of zoster [1]. However, some complications, such as postherpetic neuralgia, can also occur without predisposing risk factors [10].

Meanwhile, involvement of the maxillary or mandibular branch of the trigeminal nerve can result in oral and dental complications, and there are diverse reports in the literature describing not only osteonecrosis but also tooth exfoliation [8], ulcers, and vesicles on the oral mucosa and palate [13], localized odontalgia [14], apical periodontitis [15], periapical abscess [15], external and internal root resorption [7,16], and abnormal development of the permanent teeth [17].

A summary of the literature addressing patients with herpes zoster-related osteonecrosis of the jaw is shown in Table 1. Only articles published in English are included. All of the reports describe herpes zoster-related osteonecrosis; other herpes zoster-related oral and dental complications without osteonecrosis were excluded. Similar to other types of osteonecrosis, the mandible was more frequently involved than the maxilla (3.4:1) based on the articles shown in Table 1. In contrast to what is commonly known as “MRONJ”, it is more prevalent in women because of its underlying disease [6], while the prevalence in men was higher (1.8:1) in the retrieved articles. Another aspect of the articles reviewed in Table 1 is that all but 13 of the 63 cases had systemic diseases associated with immunosuppressive conditions. This suggests that ONJ related to herpes zoster is likely linked to immunosuppressive conditions, similar to other related complications. Treatment was mostly surgical, such as sequestrectomy or removal of the necrotic bone. In some cases, only conservative treatment was used, likely due to the small size of the necrotic bone and its spontaneous sequestration from the healthy surrounding bone. All treatments were accompanied by antibiotic therapy, except for a few cases where no treatment data were available. Due to the extensive exposure of necrotic bone outside the soft tissue and the concurrent maxillary sinus infection, surgical removal of the necrotic bone was deemed necessary in this case. Conservative management would be more suitable for narrower lesions, as mentioned previously. According to Mintz and Anavi [18], osteonecrosis occurred within approximately 21 days after the initial herpes zoster infection. However, in most previous studies, as in this case, patients already had osteonecrosis when they visited the hospital. Therefore, it is difficult to know how long it took for herpes zoster to cause osteonecrosis.

Although controversial, there are several hypotheses regarding the pathological mechanism of herpes zoster-related osteonecrosis. The most accepted is that neural inflammation caused by VZV may induce vasculitis in adjacent blood vessels [4]. Histological and immunohistochemical analysis of VZV-infected arteries revealed a thickened intima, disrupted internal elastic lamina, and loss of smooth muscle cells, which contributes to the weakening of the vessel wall, its occlusion, and subsequent tissue ischemia [15]. Based on these findings, Wright et al. [4] pointed out that tissue necrosis could be the result of ischemia of the pulp as a consequence of viral-induced vasculitis of the supplying vessels [15]. Another plausible view of herpes zoster-related osteonecrosis is that generalized infection of the trigeminal nerve is responsible for periosteal and perivascular vasculitis, leading to bone necrosis and teeth exfoliation [19]. A recent case report attempting to determine the pathogenesis of ONJ following herpes zoster infection even suggested that the coexistence of bacterial and viral infection may play a role [20]. All of these hypotheses, however, remain contentious.

Regarding ONJ, the most widely known condition until recently, MRONJ, also has uncertainties about its causes. According to the 2022 update of the AAOMS position paper, factors such as bone remodeling inhibition, inflammation or infection, angiogenesis inhibition, immune dysfunction, and genetic factors are discussed as the pathophysiology of MRONJ. Among these, bone remodeling inhibition is considered a central element. Additionally, the spread of inflammation through extraction sites might play an important role [6].

In the author’s opinion, it is difficult to differentiate between the presentation of ONJ in MRONJ and herpes zoster-related ONJ, as seen in this case, based solely on clinical aspects. However, in the present case, the author presumed that herpes zoster played a significant role in osteonecrosis of the maxilla for several reasons. First, the patient did not have any history of systemic disease or history of medication known to be related to osteonecrosis of the jaw. Second, on computed tomography and at the operation, the necrotic area exhibited a limited range in the left maxilla, while the right maxilla was essentially unaffected. Third, there was no evidence that the patient experienced a traumatic maxillary fracture that could have led to ischemic osteonecrosis before the pathological fracture occurred. Moreover, despite the limitation in mouth opening, her general oral hygiene was adequate after losing multiple teeth. Therefore, the possibility that infections through the extraction socket alone could have caused bone necrosis appears to be extremely low, especially at the maxilla, which has abundant blood supply. Therefore, it appears more reasonable that after tooth exfoliation, the VZV infection accelerated localized osteonecrosis of the maxillary bone. Incidentally, even if the patient was postmenopausal with decreased estrogen levels, which could have altered bone homeostasis [21], such an effect appears to be too weak or unclear without related systemic disease.

**Table 1:** A summary of the literature addressing patients with herpes zoster-related osteonecrosis of the jaw.

No.	Author/Year	No. of Cases	Age/Gender	Systemic Disease	Site of Necrosis	Treatment
1	Hall et al., 1974 [22]	1	62/F	Reticulum cell sarcoma (nasopharynx), radiation therapy	Maxilla	Necrotic bone removal
2	Vickery et al., 1976 [23]	1	41/F	Disseminated Hodgkin’s, stage IV-B, chemotherapy	Mandible	Decortication
3	Cooper, 1977 [24]	2	76/M 85/M	No major illness	Maxilla, Mandible	NP NP
4	Schwartz et al., 1982 [25]	1	66/F	No major illness	Mandible	Necrotic bone removal
5	Wright et al., 1983 [4]	1	56/F	Histiocytic lymphoma, stage IV-B, chemoradiation therapy	Maxilla	Necrotic bone removal
6	Garty et al., 1985 [26]	1	12/F	No major illness	Maxilla	Necrotic bone removal
7	Manz et al., 1986 [27]	1	60/F	Polymyalgia rheumatica	Mandible	None
8	Mostofi et al., 1987 [28]	1	56/NP	Leukemia, chemotherapy	Mandible	Sequestrectomy
9	Muto et al., 1990 [19]	1	72/M	NP	Mandible	Sequestrectomy
10	Mintz et al., 1992 [18]	1	50/M	NP	Maxilla	Sequestrectomy
11	Owotade et al., 1999 [29]	1	45/M	No major illness	Maxilla	Sequestrectomy
12	Pogrel et al., 2003 [30]	1	51/M	NP	Maxilla	Inferior Hemimaxillectomy
13	Arikawa et al., 2003 [31]	1	74/M	Pharyngeal cancer, radiation therapy	Mandible	Sequestrectomy
14	Yamamoto et al., 2004 [32]	1	76/M	NP	Mandible	Necrotic bone removal
15	Mendieta et al., 2005 [11]	1	63/F	No Major Illness	Mandible	Necrotic bone removal
16	van Heerden et al., 2005 [33]	6	36, 38, 47, 48, 52, 73 (gender NP)	HIV	Mandible	NP
17	Siwamogstam et al., 2006 [34]	4	30/F, 31/M, 29/M, 31/F	HIV	2 Maxilla, 2 Mandible	1 Sequestrectomy (Maxilla) 1 necrotic bone removal (1 mandible) 2 None
18	Pillai et al., 2006 [35]	1	34/M	No Major Illness	Maxilla	Sequestrectomy
19	Meer S et al., 2006 [36]	1	70/M	Diabetes, CMV infection	Mandible	Sequestrectomy
20	Feller et al., 2008 [37]	1	30/M	HIV	Mandible	NP
21	Kamarthi et al., 2009 [38]	1	43/M	HIV	Mandible	Necrotic bone removal
22	Badjate et al., 2009 [39]	1	86/M	No major illness	Maxilla	Surgical debridement
23	Jain et al., 2010 [9]	1	65/M	No major illness	Mandible	Sequestrectomy
24	Kim et al., 2012 [40]	2	78/M 77/M	No major illness	Mandible Mandible	Sequestrectomy Conservative
25	Lambade et al., 2012 [41]	1	52/M	NP	Maxilla	Conservative
26	Asha et al., 2014 [42]	1	75/M	No major illness	Mandible	NP
27	Cloarec et al., 2014 [43]	1	50/M	HIV	Mandible	Conservative
28	Rudd et al., 2014 [44]	1	59/M	Granulomatosis with polyangiitis	Mandible	Surgical debridement
29	* Tabrizi et al., 2014 [45]	30	Mean age 52.6 ± 10.6 16 Male, 14 Female	Dialysis, Chemotherapy, Transplantation	Mandible (in 21 case)	23 Surgical debridement 7 Conservative
30	Arora et al., 2015 [46]	1	58/M	HIV	Mandible	Surgical debridement
31	Patil et al., 2015 [47]	1	58/M	HIV	Maxilla	Conservative
32	Song et al., 2015 [48]	1	64/M	No major illness	Mandible	Sequestrectomy
33	Gholami et al., 2016 [49]	2	53/F 54/M	Kidney transplantation Diabetes	Mandible Mandible	Sequestrectomy
34	Faure et al., 2021 [50]	1	87/M	Myocardial infarction, Diabetes	Mandible	Surgical resection
35	Chatterjee et al., 2023 [51]	1	51/M	No major illness	Mandible	Necrotic bone removal
36	Huang et al., 2024 [20]	1	67/M	HBV		Surgical debridement
37	Present case	1	73/F	No major illness	Maxilla	Sequestrectomy

Modified and updated from Wright et al. [4], R Kaur et al. [12]. * Tabrizi et al. [45]; 21 of 30 patients suffered from osteonecrosis while others did not. NP: not provided, HIV: Human Immunodeficiency Virus infection, HBV: Hepatitis B virus infection.

## 4. Conclusions

To understand the pathological mechanism of herpes zoster-related ONJ, further research, including the role of tooth exfoliation and subsequent bacterial or viral infection, is needed. Nevertheless, the possibility of osteonecrosis of the jaw (ONJ) cannot be completely ruled out in patients with herpes zoster infection of the maxillary or mandibular branches of the trigeminal nerve. Therefore, careful monitoring of changes in the jaw, including the teeth, is necessary after herpes zoster treatment. Additionally, when encountering patients with idiopathic localized ONJ, the influence of herpes zoster virus infection should also be considered.

## Data Availability

There was no new data created, and the data is unavailable due to privacy.
